# Transition to ‘Close Care’ with a Joint Roadmap for Person-Centered and Integrated Care

**DOI:** 10.5334/ijic.10226

**Published:** 2026-10-01

**Authors:** Karin Strömqvist Bååthe, Ulrica von Thiele Schwarz, Osman Aytar, Malin Sjöqvist, Marina Arkkukangas

**Affiliations:** 1 Department of Social Science and Humanities, Mälardalen University, Eskilstuna, Sweden; 2 Research and Development Unit, FoU i Sörmland, Region Sörmland, Eskilstuna, Sweden; 3 Department of Health Care 3, School of Health and Welfare, Dalarna University, Falun, Sweden; 4 Department of Countywide Collaboration Support Unit, Region Sörmland, Nyköping, Sweden; 5 Department of Health Sciences, Innovation and Design, Mälardalen University, Västerås, Sweden

**Keywords:** collaboration, health and social service management, network theory, organizational change, relational coordination

## Abstract

**Introduction:**

Health and social services face increasing fragmentation and costs, underscoring the need to transition toward more person-centered and integrated care. The Swedish government’s ‘Close Care’ [Nära vård] initiative promotes this transition by encouraging shared regional visions and roadmaps. However, translating a vision into practice remains challenging. This study aimed to explore the experiences of healthcare and social services stakeholders regarding the transition to Close Care, focusing on using a joint roadmap.

**Methods:**

This qualitative study included three focus groups with 19 regional healthcare and municipal social service representatives, analyzed using reflexive thematic analysis.

**Results:**

Four main themes summarize the findings: 1) A rocky road toward Close Care; 2) A patchwork of Roadmap practices; 3) Navigating ambiguity: defining and positioning the roadmap; 4) Mobilizing shared meaning for Close Care and the roadmap.

**Discussion:**

A growing understanding, awareness, and engagement with Close Care is demonstrated, although challenges, including limited cross-organizational leadership and dialogue, remain. Participants reported varied familiarity and strategic document use with the roadmap, which was often considered vague or irrelevant for actionable guidance.

**Conclusion:**

Roadmap clarity, linked to measurable indicators, and strategic leadership and dialogue are required to strengthen relational coordination and transition toward more person-centered and integrated care.

## Introduction

Healthcare systems are increasingly challenged by aging populations with complex and chronic needs [1] and populations having limited and uneven access to adequate healthcare [2,3]. Providers experience difficulties in providing effective support for fragile and high-risk groups [4]. Concurrently, medical discoveries have significantly expanded treatment possibilities and improved survival rates. While these advances have enhanced patient outcomes, they have also contributed to rising healthcare costs and increasing complexity within healthcare delivery systems [5,6]. Together, these developments have exposed organizational weaknesses and challenges in coordination of care across healthcare and social service sectors. Thus, fragmented care has emerged as a central challenge, underscoring the need for more proactive and integrated systems that connect services through coordinated care chains. Over the past few decades, substantial efforts have been made to address these issues and promote integrated models of care [6,7]. In 2015, the World Health Organization (WHO) [2] launched a strategy for ‘people-centered and integrated health services,’ aiming to encourage member states to redesign their healthcare systems to better address current and future population needs through a person-centered and coordinated care chain approach. However, the implementation of this strategy faces several persistent cultural, legal, structural, and financial barriers that must be addressed to facilitate the transition towards integrated and person-centered care [1,8].

Over a decade ago, Ahgren and Axelsson [9] observed that Swedish policymakers simultaneously promoted integrated healthcare while enacting policies that fragmented services and eroded trust in collaborative efforts. While consistency is not always a political priority, such contradictory approaches may reflect the limited available evidence regarding the benefits of integrated care. Therefore, greater efforts should be devoted to evaluating integrated healthcare and related initiatives to ensure that policy decisions are consistent and guided by empirical evidence, rather than political ideology. This concern is echoed in findings from interviews across 27 European countries, which revealed that the adoption of person-centered care is influenced by national ‘*governance structures, cultural norms, and systemic fragmentation*’ [10]. Sweden is no exception to these challenges [11]. In the Swedish healthcare system, regions and municipalities are organizationally and financially independent from each other, connected solely through the citizens they serve. Figure 1 developed by the authors of this study, inspired by Ludvigsson et al. [12] and Fredriksson and Winblad [13] illustrates these findings. It shows a structural separation that causes fragmentation, exacerbating difficulties in achieving cohesive, person-centered care delivery across hierarchical levels and between organizations operating at the same level.

**Figure 1:**
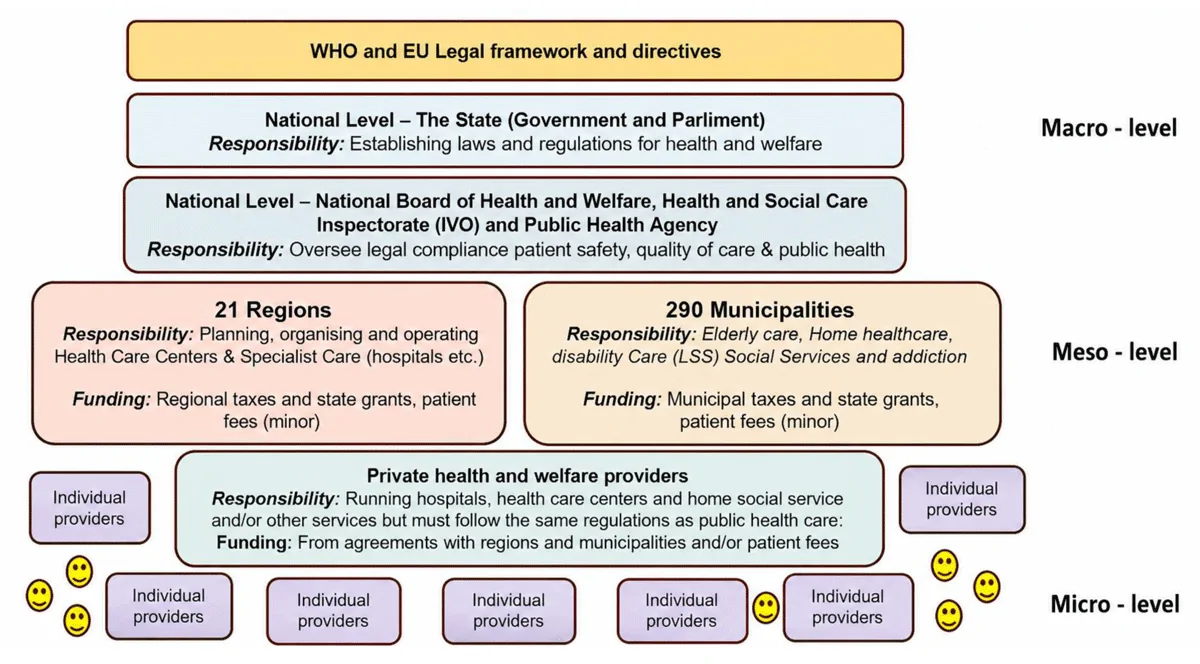
The governance model of the Swedish health and welfare system.

Person-centered and integrated care often involves multi-organizational collaboration at the micro, meso, and macro levels, presenting crucial challenges [14]. Although the macro level (national and international policy) and the micro level (clinical practice and patient care) have been extensively studied, the meso level has received comparatively limited attention. The meso level is particularly important in highly decentralized healthcare systems, as it involves the coordination between regional and municipal actors responsible for translating national policy requirements into integrated health and social care practices. In Sweden, this coordination responsibility is especially pronounced, since regions and municipalities hold substantial autonomy but are jointly responsible for organizing and delivering health care and social services. As a result, the meso level becomes vital for understanding how integrated care is implemented and coordinated in practice across organizational boundaries. In Sweden, the structural setting cannot be easily altered because stakeholders belong to different responsible authorities, which, in turn, are governed by independent local political bodies and funding schemes. Instead, changes in such systems may be understood through network theories, such as relational coordination [15]. Relational coordination has been studied for approximately two decades and provides a valuable framework for examining networks, shared goals, and communication as mechanisms for integration; beyond purely structural solutions. Nevertheless, it remains underutilized in the context of integrated care [15].

### Competing logics in public health systems

The Swedish public health system is heavily influenced by two potentially conflicting governance paradigms, managerialism and professionalism [13,16], influencing the actors expected to collaborate in transforming Swedish health and social services into a more person-centered and integrated model of care.

Before the 1990’s and throughout most modern medicine licensed professionals in Sweden and in most countries of the Organization for Economic Cooperation and Development (OECD) have had a large autonomy in practicing their professions in health care [16]. As a governance paradigm, professionalism builds on the unique and intrinsic knowledge of professionals, collegial cooperation and joint learning as a foundation for organizing work [17,18]. However, professionalism has been affected by New Public Management [19] introduced in Sweden in the 1990’s [13,16] as it brought about managerialism as a guiding principle. New Public Management emphasizes the application of business principles to public sector organizations, with a focus on efficiency and accountability [19]. In this paradigm, managerial bureaucracy becomes an organizational structure that enforces managerial control. In these bureaucratic settings, decisions are made based on performance metrics and adherence to standardized procedures, reinforcing the goals of managerialism [13,16].

Currently, managerialism and professionalism co-exist in the Swedish health and social care management setting. However, trying to operate under these two paradigms simultaneously or in a hybrid format generate tension. Bureaucratic systems rely on rules and procedures, and managerial reforms push for measurable outcomes and often constrain professional discretion. This tension complicates consensus building and obscures shared goals and risks, producing fragmented rather than coordinated strategies for integration [16,20,21]. A structured roadmap might mitigate these risks by providing a common framework for negotiation of tasks, responsibilities and collaboration.

### Contextualizing the study

The Swedish Government Investigation *Good and Close Care* [22] highlighted the urgent need for healthcare system transformation. In Sweden, person-centered and integrated care are jointly conceptualized as *Close Care*, which is the term used throughout this study. Although not exactly the same, the Swedish Close Care concept aligns with the WHO perspective that person-centeredness is an integral component of integrated care [23].

Several initiatives have supported the implementation of Close Care in Sweden. Following the introduction of a national vision, the Swedish Association of Local Authorities and Regions (SALAR) encouraged all 21 healthcare regions to develop Close Care roadmaps to translate strategic visions into concrete local actions and plans. Roadmaps are commonly used to bridge long-term visions and implementation plans, helping organizations move beyond short-term goals [24]. However, the usefulness of operational roadmaps in health and social service management has rarely been investigated.

In 2022, a Participatory Action Research (PAR)-inspired project was initiated in the region of Sörmland, Sweden, to support the transition toward Close Care in healthcare and social services with the aim of finding new ways of working more proactively and together across organizational health care and social service boundaries. This integration effort involved one region and nine municipalities. While developing a shared vision broadened our understanding of the concept, it offered limited structural or operational guidance, leaving the transition fragmented and weakly supported [25]. Despite the regional vision, system-level changes have been limited, partly owing to Sweden’s highly decentralized tax-funded system of 21 regions and 290 municipalities [10,26]. The roadmap tool was then introduced by SALAR and expected to function as a strategic planning instrument and basis for allocating grants to Close Care initiatives. Therefore, this study aimed to explore the experiences of healthcare and social services stakeholders regarding the transition to Close Care, focusing on the use of a joint roadmap.

## Methods

### Study design

This qualitative study was part of a larger project that used a practice-oriented research approach inspired by PAR [27]. This methodology emphasizes inclusive and equitable collaboration between researchers and practitioners, enabling joint exploration and contribution to the transition, while continuously supporting and learning from the process. The utilized qualitative method was dialogic, aiming to co-produce knowledge regarding the status of regional and municipal transitions to Close Care. The main data sources for this study are focus groups. However, to provide contextual understanding of the implementation process, supplementary background material was collected and reviewed. This included meeting notes from the Close Care Network, whose members meet monthly, as well as official reports [28,29] and notes from dialogue meetings between researchers and the municipal and regional representatives.

### Participant recruitment

Participants were recruited through purposive sampling and included individuals who had either contributed to the development of, or were actively engaged in, the regional Roadmap for Close Care.

Recruitment was conducted through presentations by a regional representative and the first author who introduced the study and invited participation. An open invitation was first extended to members of the Close Care Network, comprising key stakeholders involved in the reform from all nine municipalities and the regional organization. These stakeholders included municipal Close Care process leaders and regional representatives from primary care, psychiatry, medical services, hospital coordination, regional support functions, and proactive health coaching (n = 18 invited).

A second presentation and invitation were directed at the Regional Steering Group (n = 16), a management network established to coordinate collaboration between the Region and municipalities, as well as between health care and social services.

Nineteen individuals volunteered to participate and were allocated to three focus groups: one consisting of Regional Group managers, one of municipal representatives, and one of regional representatives. All nine municipalities and the major organizational functions involved in the Close Care initiative were represented. Given the roadmap’s purpose of providing a shared direction for integrated care, particular attention was paid to ensuring representation from both strategic and operational levels, including the Regional Steering Group as well as regional and municipal stakeholders expected to collaborate in addressing citizens’ health and social care needs. Participants in the focus group included Close Care Coordinators, Service Development Managers, Physicians, Directors of Social Services, Hospital Coordinators, and Local Process Leaders, Service Development Managers, Physiotherapists, and Unit Managers. To protect participant confidentiality, quotations are presented collectively and identified only by participant numbers (1–19), without reference to profession, role, or group affiliation.

### Procedure and data collection

The three focus group interviews were conducted online to maximize accessibility and facilitate participation regardless of geographical location. Data collection took place during May – July, 2024. Each focus group lasted between 55 and 75 minutes and included 5–8 participants.

The focus group interviews were conducted in Swedish by the first author in collaboration with the regional process leader for Close Care, who is also a co-author of this study. Conducting the interviews jointly enabled the incorporation of contextual knowledge throughout the data collection process while supporting a shared interpretive understanding of the topic under investigation.

The discussions were guided by the semi-structured interview guide, which was informed by previous project work, study aims, and relevant background documentation. Each interview began with open-ended questions exploring participants’ perceptions of the current status of the transition toward Close Care within their respective organizations. This introductory discussion provided contextual grounding and facilitated subsequent reflections on the roadmap as a tool. The remainder of the interview focused on participants’ experiences of using the roadmap, perceived barriers and facilitators to its implementation, and suggestions for improving its usability and relevance in practice.

### Data analysis

The focus group interviews were recorded, transcribed verbatim, and analyzed using reflexive thematic analysis, following the six-step process outlined by Braun and Clarke [30,31] and further elaborated on by Byrne [32]. The phases were as follows: 1) familiarization with the data, 2) initial coding, 3) generating candidate themes, 4) reviewing and refining themes, 5) defining and naming themes, and 6) reporting the findings to all relevant stakeholders in group dialogues.

Thematic analysis was selected because it enables the active involvement of participants in the data analysis process [30] aligns with the collaborative and reflective nature of PAR, in which the co-construction of meaning is central. Additionally, thematic analysis offers flexibility in capturing the social and psychological dimensions of participants’ experiences, rendering it adaptable to a wide range of epistemological and theoretical frameworks [30]. Its capacity to generate rich, nuanced insights supports the development of analyses that can inform policymakers and drive change, and it is particularly valuable in healthcare and social research owing to its accessibility and relevance across disciplines [31].

The adopted reflexive analytical approach recognizes the active role of researchers in generating meaning. Interpretations of the themes were shaped by individual researcher reflections and group dialogue during and after data collection. The analysis aimed to capture shared patterns and variations in participants’ experiences of the roadmap and provide suggestions for improvement in relation to the transition toward Close Care and system-level changes through the implementation of a roadmap.

First author and one of the co-authors independently familiarized themselves with the data by reading through the background material and the transcripts from the focus groups (step 1). They then performed the initial coding (step 2), followed by the clustering of codes and generation of candidate themes (step 3). After comparing the coding results and candidate themes they collaboratively reviewed and refined the candidate themes (step 4), resulting in 12 final themes organized under four main themes. In line with the principles of PAR, the findings were further developed and discussed through “learning loops” involving interview participants; and members of the regional Close Care network. This process included two reflection and feedback sessions designed to inform and work together on step 5 in the Braun and Clarke RTA methodology: Defining and naming themes. During this session the fourth theme of “*Mobilizing shared meaning for Close Care and the Roadmap use through relational coordination*” was adjusted to cut across the other three themes. Additional discussions occurred during step 6 in the RTA methodology [31,32] during a digital Close Care Network Meeting in the autumn of 2024, where findings were presented for large-group dialogue, fostering co-interpretation and shared ownership of the results [27]. Furthermore, the initial findings were presented and confirmed in the Regional Steering Group in the spring of 2025. Thereafter, the identified themes were further discussed and refined by first author and regional representatives, followed by last author, and a final consensus was reached between all authors of this article (who all had read the background material and transcripts before the final agreement).

### Lived experiences

The development of the vision and roadmap was grounded in a series of participatory workshops involving both health and social care professionals, as well as representatives from citizen organizations, including retirement associations and groups representing individuals with medical challenges. To ensure that the study reflects authentic, practice-based insights, particular emphasis was placed on capturing the lived experiences of health and social care professionals actively engaged in the transition toward Close Care through cooperation and collaboration between different departments and professions. These professionals’ first-hand accounts and reflections provided critical input into understanding the practical realities, challenges, and opportunities associated with implementing integrated care at the local level. Their experiential knowledge served not only as empirical material but also as a guiding perspective in shaping the recommendations.

This study was approved by the Swedish Ethical Review Authority (approval no. 2023-00064-01) and was conducted in accordance with the principles embodied in the 2024 update of the 1964 Declaration of Helsinki. Verbal informed consent was obtained from all study participants.

## Results

Participants’ experiences of the Close Care transition guided by the roadmap were shaped by multiple interconnected factors. In total, four main themes and 12 subthemes were developed, as shown in Figure 2.

**Figure 2:**
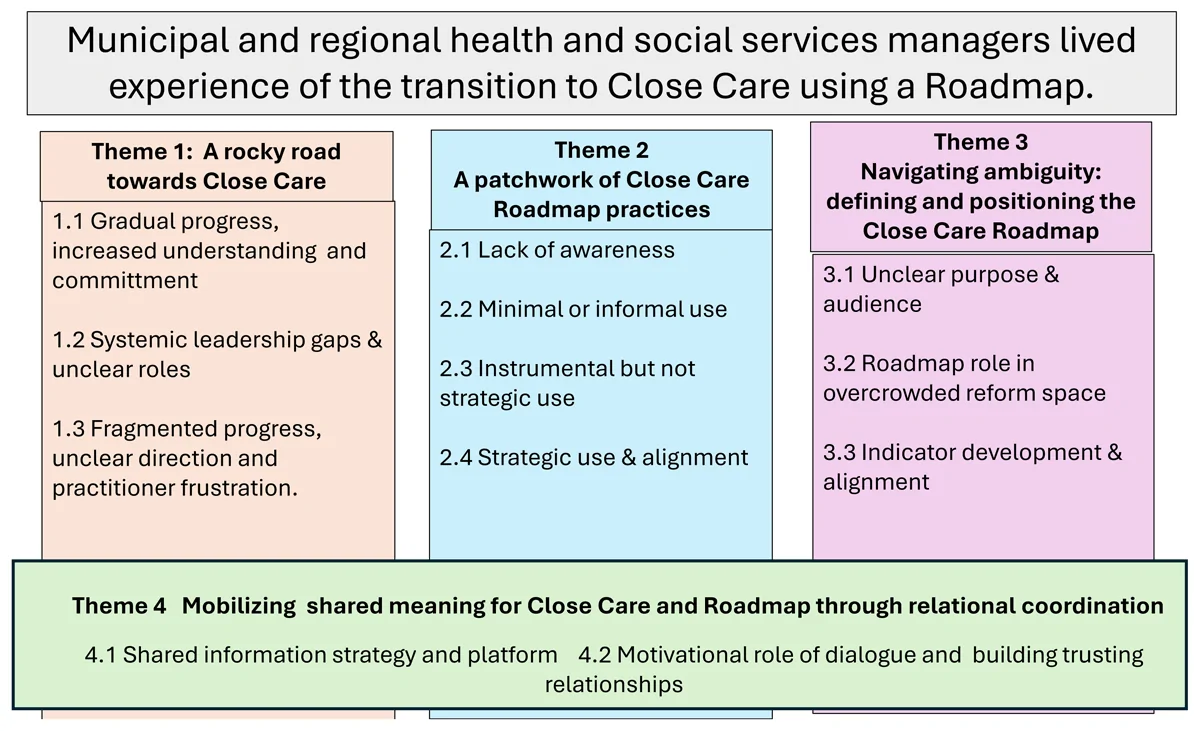
Presentation of 4 main themes and 12 subthemes according to thematic analysis based on Braun and Clarke (2006, 2014).

Four themes were generated from the analysis. The first theme, *A Rocky Road Towards Close Care*, captures participants’ experiences of gradual understanding of the Close Care concept coupled with fragmented progress and gaps in systemic leadership. The second theme: *A Patchwork of Close Care Roadmap Practices* describes variations in engagement with the recently developed roadmap across individuals and organizational contexts. The third theme: *Navigating Ambiguity Defining and Positioning the Close Care Roadmap* concerns perceptions of the roadmap’s strategic value and opportunities for its further development. The fourth theme, *Mobilizing Shared Meaning for Close Care and the Roadmap* focuses on communication, shared understanding, and dialogue as central elements of implementation and collaboration and cuts across the three first themes as it was expressed by participants to be imperative as a catalyst for adapting both the concept of Close Care and the roadmap as a tool.

### Theme 1: A rocky road towards Close Care

The first theme presented the participants’ lived experiences of the ongoing transition towards Close Care and social services in general. This theme illustrates how the transition phase is not merely slow but marked by a fluctuating pace, loss of focus, and emotional fatigue.

#### Subtheme 1.1: Gradual progress and increased understanding and commitment to Close Care

The participants described a growing understanding and acknowledgement of Close Care although implementation remains slow. Several municipal representatives emphasized increasing internal support within their municipalities; nevertheless, they stressed the need for increased collaboration with the region. Alongside these developments, a sense of cautious optimism emerged throughout the discussions. The focus group dialogue itself was described as energizing and motivating, suggesting that engaging in collective reflection fosters renewed commitment and enthusiasm. Despite ongoing challenges, the participants expressed a strong sense of dedication to the principles of Close Care.

*‘We try to keep Close Care in mind… especially in our change work.’* (Participant 13)

*‘We have at least made a project portfolio here in our municipality for person-centeredness, so there we have a plan.’* (Participant 7)

*‘With the new social services law, Close Care will join forces—we see Close Care as a ‘perspective’… now we can get support from the law.’* (Participant 5)

#### Subtheme 1.2: Systemic leadership gaps and unclear roles

Participants, particularly those from municipal settings, emphasized the lack of clearly defined roles and mandates in their function as process leaders. These individuals are expected to lead and facilitate major transformation processes without authority, structural support, or integration into formal governance frameworks. Consequently, they felt that their ability to drive meaningful change was constrained and described a sense of being positioned outside the core decision-making structure, further limiting their capacity to effectively act. In addition, the participants reflected on the disconnect between the creation of such roles and the absence of adequate operational support.

*‘I am not in the right forum within my organization; it is difficult to find the appropriate contacts for follow-up … If I as a coordinator should be the engine for this transition it does not work if I’m missing the wheels and the hood.’* (Participant 7)

*‘We tend to create roles, but we cannot always provide the conditions for them to succeed.’* (Participant 18)

*‘We have the Regional Steering Board, the Network for Close Care, projects to asses health care routines, discharge processes, emergency processes and all these processes here and there, but all these actions we are tasked with needs to come from one place and common thinking/strategy and then reach the whole operations and get closer to the patient to give an effect…right now it’s all over the place.’* (Participant 3)

These structural misalignments were further compounded by resource constraints at both the regional and municipal levels, which the participants described as draining momentum from the transition. The participants called for a stronger strategic direction and renewed their commitment to support the process.

*‘The transition has not really reached the operational level yet — it needs to trickle down into the organizations.’* (Participant 3) *‘We could use a fresh start: a boost.’* (Participant 14)

Furthermore, the participants expressed a lack of “system-level leadership,” highlighting fragmented governance, unclear reporting lines, a sense of disconnection from decision-making bodies, and perceived gaps in strategic guidance from central coordinating bodies.

*‘There’s perspective overload… and it’s hard to reach the system leadership level… it’s like a wet blanket over the whole collaboration structure.’* (Participant 5)

*‘We do not always have the guidance we need from the Regional Steering Group, there are no clear priorities.’* (Participant 17)

Finally, the participants underlined the need for more visible and committed political leadership to provide both direction and legitimacy to the transformation process.

*‘We hoped for a clearer and more committed stance from political leaders to energize the transformation.’* (Participant 7)

#### Subtheme 1.3: Fragmented progress, unclear direction, and practitioner frustration

Participants experienced the transition to Close Care as uneven, fragmented, and difficult to sustain. They described it as uncertain and intangible, with slow, inconsistent, and sometimes cyclical progress. Ambiguity, disjointed processes, and perceived contradictions in how progress was reported contributed to frustration, disillusionment, and diminished motivation, reinforcing the sense that strategic ambitions did not translate into tangible or actionable change.

*‘The National Board of Health and Welfare’s follow-up reports have not been very positive… But SALAR’s annual reports are more optimistic…’* (Participant 13)

*‘It’s a little bit like an accordion. We join forces [between organizations and professions] and then we let go again and then we go forward and then backward again.’* (Participant 1)

*‘This stepwise movement – it’s more like ant steps’* (Participant 19)

The participants described the transition as frustrating, slow and hard to understand.

*‘Our reality is tougher than two years ago… we collaborate less despite that we talk MORE about it.’* (Participant 1)

*‘We ignore the individual patient — this creates great frustration among health care providers… trying to support and find a solution for a patient… only to be met with a cold shoulder.’* (Participant 2)

*‘We might want to move faster, but I feel like we are out of sync—there’s an uneven pace between municipalities and regions.’* (Participant 9)

*‘Close Care is a transformation that—well, I do not want to say it, but it feels like an amoeba. It’s hard to get a grip on it.’* (Participant 12)

### Theme: 2. A patchwork of roadmap practices

Participants reflected on how the roadmap was used in practice, ranging from complete unawareness or non-use, limited or informal application, instrumental use as a flexible guide, to full strategic integration, where the roadmap actively shaped municipal activities and clarified joint agreements.

#### Subtheme: 2.1 Lack of awareness and/or engagement

Participants indicated that the roadmap was largely unknown or unused within their organization, with some expressing a sense of embarrassment regarding this. Certain participants did not consider non-use of the roadmap to be an issue in their local contexts.

*‘In my supporting role to the division management I have not used the roadmap actively, and if I may “swear in church”, I hardly even knew that it kind of existed until sometime last spring.’* (Participant 2)

‘*There are many activities that are difficult to get a handle on. I haven’t found anyone here who has even heard of it, and then it becomes very difficult to stand as responsible for something in the roadmap when we don’t even know what it is.’* (Participant 7).

#### Subtheme: 2.2 Minimal or informal use

Participants described limited or individual use of the roadmap, suggesting a more informal engagement, such as using it as a point of reference for activities.

*‘But when it comes to the activity level, well, this Roadmap has helped in my work, you could say as a kind of reference document, to see which areas are being prioritized within the county … but not in the sense that we sit down with it and say, OK, which activities are we doing here connected to it.’* (Participant 18)

#### Subtheme 2.3: Instrumental but not strategic

The participants expressed that some municipalities used the roadmap as a foundational or interpretative tool, without formally embedding it into their strategies and that they see the roadmap as a more flexible guideline than a mandatory framework.

*‘I don’t let the roadmap take such a big place in the transition to Close Care. If you read the government assignment, the big one, it is a larger transition — healthcare is just a small slice of the pie… you mustn’t get stuck focusing only on healthcare.’* (Participant 5)

*‘We see I as a basic document to build on with own activities.’* (Participant 10)

#### Subtheme 2.4: Strategic use and alignment

At the highest level of engagement, the participants considered the roadmap as a central document guiding municipal activities. The participants noted that it clarified joint agreements and served as a supporting document. These responses suggest that the roadmap is embedded in organizational strategies and practices.

*‘We have used the roadmap as a basis when developing all our activities for Close Care.’*(Participant 6)

*‘The Roadmap gives us clear guidance on which activities to focus.’* (Participant 7)

### Theme 3: Navigating ambiguity: defining and positioning the Close Care roadmap

Participants expressed concerns about the regional roadmap’s ambiguity and limited practical utility. Many were uncertain about its purpose and application, describing it as abstract and disconnected from daily work. However, they recognized its potential to support indicator development and progress monitoring if its purpose and target audience were clarified.

#### Subtheme 3.1: Unclear purpose and audience

A recurring issue expressed by the participants was the lack of clarity around who the roadmap was intended for. This ambiguity appeared to hinder its adoption, as staff across different levels of the organization were unsure of its relevance to their roles. Some questioned whether the roadmap was designed as a high-level strategy for leadership, a planning tool for process managers, or as a guide for frontline implementation. The document remains underutilized without a clear target audience or purpose.

*‘Who is the roadmap actually for? Managers? Process leaders? Frontline staff?’*(Participant 8)

*‘Yes, but what is actually happening? Who is responsible for following up the different parts, how far have we come?’* (Participant 1)

*‘Is this a document that should just show the roadmap and then not have any activities at all connected to it, or what do we want…what is the actual use? What results do we want to accomplish with this Roadmap?’* (Participant 8)

*‘My experience of this Roadmap is confusion and it’s not helping us forward.’* (Participant 4)

#### Subtheme 3.2: Roadmap’s role in an overcrowded reform space

In the discussions about the plethora of initiatives in the transition to a more person-centered and integrated care, participants strongly expressed that documents such as the roadmap should help staff see the “red thread” throughout the initiatives. One of the most prominent desires was for the roadmap to better illustrate the overarching logic of the transition, offering what several participants described as a visible “red thread” connecting various initiatives. Without such a thread, staff members were perceived as struggling to navigate or prioritize their efforts.

*‘We need to help our staff to see the red thread.’* (Participant 9)

This call for coherence reflects the need for strategic documents, such as the roadmap, to function as aspirational frameworks and practical tools that guide implementation in concrete terms. Participants described how the breadth and vagueness of the roadmap could dilute its effectiveness and render it abstract rather than actionable.

*‘I believe the biggest challenge or complexity is the width of this, that everything is Close Care… it [roadmap] feels like it’s the next years’ plan for social services in its entirety.’* (Participant 8)

In this context, the participants viewed the roadmap as a potential tool to more clearly prioritize and sequence initiatives. Participants emphasized the importance of explicitly linking the roadmap to relevant legislation, such as the revised Social Services Act (SOL). They argued that such links would lend legitimacy and policy alignment to the work and render the document more relevant to daily operations.

*‘… it does not have to be a lengthy text but it should be mentioned in a few sentences how it connects to SOL.’* (Participant 5)

However, the participants recognized that clarifying the roadmap’s purpose and aligning it with law and practice would not be sufficient on their own. They argued that what was ultimately needed was a broader systemic transformation that included rethinking ways of working, leadership structures, and how change was supported and governed across organizational levels.

*‘One must change not only the roadmap but also the ways we are working. What do we need to achieve systemic change with systemic leadership?’* (Participant 2)

#### Subtheme 3.3: Indicator development and alignment

While existing indicators were perceived by the participants as missing or disconnected from the roadmap’s outlined activities, they identified this misalignment as a potential catalyst for improvement. Participants expressed a strong interest in using the roadmap as a foundation for building more relevant, activity-linked indicators that could help track and communicate progress more effectively. Therefore, the current shortcomings of the roadmap are viewed as an opportunity for collaborative development and refinement.

*‘How can we measure this? Indicators could be a winning concept’*. (Participant 1)

*‘We have an opportunity now to build a number of indicators that are directly tied to the activities outlined in the roadmap.’* (Participant 6)

*‘I see almost like a spreadsheet…less words…an operational plan.’* (Participant 17)

### Theme 4: Mobilizing shared meaning for Close Care and Roadmap through relational coordination

This theme captures the essential role of communication in enabling a system-wide transformation toward Close Care. Participants repeatedly emphasized that change efforts are unlikely to succeed without a shared understanding and coordinated messaging across all organizational levels. Communication was not simply described as a logistical need but as a foundational process for mobilizing engagement, building trust, and creating alignment among diverse actors. A recurring concern was the lack of a unified communication strategy for Close Care and the roadmap.

#### Subtheme 4.1: Need for a shared information strategy and platform

The idea of a centralized communication infrastructure, such as a webpage or knowledge hub, has been repeatedly expressed as a practical step toward strengthening shared understanding.

*‘Having a central, shared webpage could serve as a reliable reference point and help spread clear and unified messages.’* (Participant 11)

*‘I really miss a coherent information strategy from the leadership*. (Participant 16)

#### Subtheme 4.2: The motivational role of dialogue and building trusting relationships

Beyond structured communication tools, the participants emphasized the energizing effect of face-to-face dialogue and peer exchange. Informal conversations, networking, and cross-functional meetings are critical for motivation, solidarity, and resilience, particularly when change is slow or fragmented.

*‘Dialogue gives us energy, even when the process feels slow and frustrating.’* (Participant 4)

*‘Where is the captain on this ship guiding us?’* (Participant 2)

## Discussion

This study aimed to explore the experiences of healthcare and social services stakeholders regarding the transition to Close Care focusing on the use of a joint roadmap.

The first three themes describe experiences including both opportunities and barriers, the first relates to Close Care as a process while the second and third pertain to the roadmap. The fourth theme, ‘Mobilizing shared meaning through relational coordination’ was defined as a prerequisite for overcoming the current barriers identified in this study.

Without trust, dialogue, and shared communication platforms, this complex reform will remain slow, frustrating, and fragmented, falling short in meeting its original aim of transitioning to Close Care. The Swedish healthcare system has long pursued digital initiatives to improve the efficiency of health and social care processes. However, digitalization has often been approached as an end in itself or as a primarily technical undertaking, rather than as a tool for strengthening relational coordination among healthcare professionals [33]. The roadmap could act both as a symbolic and functional digital anchor reinforcing the idea that all actors are part of a joint endeavor and work form the same foundation.

The participants also noted that the absence of consistent messaging created confusion, undermined cohesion, and caused difficulty for professionals to see how their work fits into a broader transformation. Contextual documentation has reported that the aim of Regions and Municipalities developing and engaging with a vision and a joint roadmap enhances understanding of Close Care and facilitates learning among process leaders and stakeholders [29,34,35]. However, participants in the focus groups expressed frustration regarding slow and fragmented progress and difficulties in understanding and using the roadmap and provided a few concrete examples of how the roadmap directly guided operational decisions during a transition. Overall, roadmap usage ranged from nonengagement to full adoption, reflecting varying levels of awareness, relevance, and organizational capacity across municipalities and regions. This spectrum illustrates the varying degrees to which Close Care and/or the roadmap slowly have been operationalized and embedded within organizational practices. These findings echo those of the study on the Close Care vision for a Region [25] and the national evaluation in 2025, indicating a slow prognosis for Close Care implementation in Sweden [26].

While both the development of a shared vision and, more recently, the introduction of a national roadmap have contributed to a broader understanding of the Close Care concept, they have fallen short in providing the concrete structure and operational guidance that regional and municipal actors consistently request. Therefore, the transition toward more integrated and person-centered care remains fragmented and insufficiently supported, limiting its potential impact on citizens’ care experiences and outcomes. These findings align with international evidence highlighting the inherent challenges and complexities involved in implementing person-centered and integrated health and welfare reforms [10,36,37].

Furthermore, from a relational coordination lens [15] the implementation of the Close Care reform is complicated by unclear network structures and different management perspectives within and between organizations and professions. While the “person-centered” approach often relies heavily on a professionalism perspective [38,39], the “integrated care” part of Close Care is often approached through a managerial lens. These logics often pull in different directions. Managerialism, and the bureaucracy that often accompanies integrated care plans, typically relies on a top-down approach using tools such as key performance indicators, standardization, and audits, which can easily feel misaligned with the bottom-up professionalism that operates through the relational, flexible ethos of “person-centered” care [18,38]. These tensions are reflected in our findings: health and social care professionals feel underinformed and disempowered by imposed structures, while managers simultaneously perceive professionals as resistant to change or hard to reach with communication efforts. Managerial systems tend to ask, “Who is responsible for what,” with clear lines of responsibility, which creates tension in the new paradigm of Close Care where shared accountability, blended roles, and collaboration across organizational and professional boundaries are emphasized [16,20]. Participant quotes highlight how participants make sense of the Close Care transformation through metaphors and narrative; describing it as “ant steps,” an “accordion,” or even an “amoeba.” These images express difficulties in following substantial forward progress or clear direction.

Furthermore, the findings indicate that the roadmap, despite its intended strategic function, has had limited influence. Many respondents were unaware of its existence, while those familiar with it often perceived it as insufficiently clear or anchored to support shared understanding, strategic alignment, and sustained momentum for Close Care implementation. Without mechanisms for grounding the roadmap in local practice, an ambiguous roadmap in the transformation risks stalling or becoming symbolic rather than substantive [24].

Our stakeholders’ interpretation of the Close Care process and roadmap aligns with the broader literature on health system reform, in which management and frontline professionals often experience change in processes as uneven, uncertain, and emotionally taxing. Moreover, it reinforces the importance of clarifying strategic goals and fostering shared meaning, management philosophies, and collaborative continuity across the system levels [6,8,22]. The participants highlighted the importance of aligning the transition to Close Care with existing national initiatives, such as the Kloka Kliniska val [Wise Clinical Choices] [40]. Research has suggested that top-down approaches often fail in complex healthcare systems and does not necessarily foster interprofessional collaboration [1,41]. Nevertheless, Sweden’s bottom-up model, in which regions and municipalities develop their own visions and roadmaps, can be slow and insufficient without strong national support structures and more direct guidance. [26,36]. Consistent with this, robust governance models are essential before meaningful system transformations occur [37].

### Strengths and limitations

We consider the practice-oriented, participatory design of the overall project to be a key strength, fostering contextual relevance and collaborative knowledge production with stakeholders who are actively involved in the transition to Close Care. The inclusion of participants from all municipalities at both regional and municipal levels enhanced the credibility of the findings. The dialogic focus group format supported rich, reflective discussions, whereas the use of multiple data sources strengthened validity through triangulation. Furthermore, although the findings are context-specific, they are likely to be broadly transferable to other regions operating within highly decentralized health and social care systems.

Nevertheless, this study has some limitations. The direct applicability may be limited to more centralized systems where governance structures and implementation contexts significantly differ. The digital interview format, while accessible, may have limited the depth of interaction. Another limitation is that the perspectives of end users, patients, and citizens were primarily interpreted and conveyed by managers and other professionals rather than directly captured. End-user involvement was considered less meaningful at this early stage, as the transition to Close Care had not yet reached frontline practice. However, end users contributed to the development of vision, as reported and published in our previous study [25]. With many unrealized changes, patients and citizens have likely seen minimal tangible differences in care, limiting their ability to assess the impact of the roadmap at this stage. The involvement of insider collaborators, including the regional process leader/coordinator and co-author was approached reflexively throughout the research process. This may introduce an interpretative bias. However, in line with Braun and Clarke’s reflexive thematic analysis [30,31] the researchers acknowledged that knowledge production and theme development are inherently interpretive and shaped by the perspectives, positions, and interactions of those involved in the study. The participation of insider collaborators contributed valuable contextual and organizational insights. One of the authors, MS, participated as a co-author through her role in practice, in line with the PAR approach, contributing to her experience of working with stakeholders involved in the transition toward Close Care in this specific context.

### Practical recommendations

Several recommendations to enhance the clarity and utility of the roadmap emerged from the stakeholder dialogue. Process goals and tools for transition, such as staffing and digitalization, should be framed as enablers, rather than end goals. Each activity on the roadmap should be linked to concrete indicators that are regularly monitored and reported by designated responsible parties to ensure that the document remains actionable. Activities should be reviewed to identify opportunities for removal, sharpening, or prioritization, with clear points of contact assigned to each organization. A comprehensive communication strategy is essential to articulate a vision and roadmap for all stakeholders. The active involvement of frontline workers and end users early in the process ensures that their lived experiences inform the development, implementation, and evaluation of integrated care.

A key prerequisite for successful implementation is the presence of a systemic and holistic leadership structure. At the same time, management must recognize and support sufficient maneuvering space for professional discretion, enabling staff to deliver person-centred and integrated care while maintaining motivation and engagement. Shared visions and roadmaps can serve as important tools for clarifying collective goals and defining what is to be achieved. However, greater autonomy should be afforded to frontline staff regarding how these goals are accomplished. In this context, managers should primarily act as enablers who create the conditions for high-quality care and shared follow up structures, rather than as controllers who prescribe processes and decisions.

## Conclusion

This study demonstrated that despite participants’ engagement and motivation, the transition to Close Care was hindered by insufficient structural support, fragmented leadership, and unclear governance. The transition was often slow and disjointed, with the roadmap offering limited guidance beyond articulating vision. The participants highlighted the need to strengthen leadership, develop clear indicators and organizational networks, and enhance communication and dialogue. Interpreted through the lens of relational coordination, these findings suggest that fostering shared goals, knowledge, respect and flexibility among stakeholders is critical for overcoming fragmentation, aligning managerial paradigms, and sustaining change.

Future research should investigate the potential of implementing Close Care and tools such as roadmaps to facilitate relational coordination and bridge conflicting managerial paradigms, thereby enhancing the effectiveness of integration change processes in fragmented healthcare systems.
